# Treasure of the Past: I: RECOMPARISON OF THE UNITED STATES PROTOTYPE METER.

**DOI:** 10.6028/jres.105.034

**Published:** 2000-04-01

**Authors:** Louis A. Fischer

Through the courtesy of Dr. Benoît, Director of the International Bureau of Weights and Measures, advantage was taken of a visit by the writer to Paris in October, 1903, to compare U. S. Prototype Meter No. 27 with the standards of the International Bureau.

## U. S. Meters Nos. 27 and 21

U. S. Meter No. 27, like the. prototypes of all the principal nations, and also like the international meter, is composed of 90 per cent platinum and 10 per cent iridium, with minute traces of other metals which compose less than 0.1 per cent of the total. It was intercompared at the International Bureau of Weights and Measures, in 1888, with the national prototypes above referred to and with the international meter; and shortly afterwards it was brought to this country by a special messenger, who certified that it had suffered no violent mechanical or temperature disturbance in transportation. Soon after its arrival in this country—or to be exact, on January 2, 1891—the standard was unpacked with considerable ceremony at the Executive Mansion in the presence of the President of the United States, who accepted it as the national prototype meter.^a^

It was then immediately repacked, sealed in its metal case, and taken to the Office of Standard Weights and Measures, in the custody of which it remained until the formation of the Bureau of Standards, on July 1, 1901, when it was transferred to the Bureau with the other apparatus belonging to the Office of Standard Weights and Measures. It remained packed and sealed in its case until a few weeks before it was taken to Europe, when it was compared wjth Meter No. 21, which is exactly similar to No. 27, except that the lines and surfaces are not as perfect as those of No. 27, on account of its having been frequently packed in shaved ice.

## Comparison of No. 27 with No. 21

This comparison was made solely to furnish a check on the length of No. 27 in case it should meet with accident in transporting it to and from Europe.

The comparisons were made on an improvised comparator installed in the subbasement of the Butler Building, in Washington, now occupied by the Bureau of Standards.

### The comparator

The comparator, which is a temporary structure, differs essentially from those used at the International Bureau of Weights and Measures, and elsewhere, and in consequence the results at present obtained with it are not as concordant as the results obtained at the international bureau. For general purposes, however, it offers decided advantages, and if properly constructed, it is believed that it will give results just as concordant.

The essential features of the comparator are two brick piers, A A, which support the ends of a heavy iron I beam, B, to which are clamped two heavy iron brackets, C C. These brackets support the microscopes D D. The microscope supports may be clamped anywhere on the I beam, and hence the comparator may be used for comparing bars having any length from 0.1 meter to 1 meter. Where the microscopes are fixed to the piers it is only possible to compare bars of a definite length. By properly protecting the I beam and microscopes against temperature changes, or by making them of the 36 per cent nickel-steel alloy, it is believed that the distance between the microscopes will be as constant, or more so, than the distance between microscopes mounted on independent piers. In addition to the I beam and its two supporting piers, two intermediate piers, E E, support steel rails upon which the carriage F moves transversely to the I beam. Mounted upon this carriage is a wooden box G covered with sheet copper, inside of which is a heavy sheet brass box in which the two bars to be compared were placed. The object of this arrangement was to secure uniform temperature within the inner box. Since the coefficients of expansion of the two bars were almost identical, an accurate knowledge of the true temperature was not important, though it was important that both have the same temperature. The box rested upon three adjusting screws used to focus the bars under the microscopes.

To protect the I beam and the microscopes and clamps from the heat of the observers’ bodies they were covered with sheet asbestos.

### Thermometers

The temperatures of the two bars were determined by means of two Tonnelot thermometers previously studied at the International Bureau of Weights and Measures. One was placed upon each bar, the bulbs being placed in opposite directions, and the thermometer scales being read through small openings in the brass and wooden inclosing boxes. As before stated, the question of the actual temperature was not of importance, because the expansions of the two bars were so nearly equal that an error of a whole degree in reading the temperature introduced an error in the final result of less than 0.01*μ*. Should, however, the temperature of one bar differ from that of the other by 0.1° it would, if not corrected for, introduce an error of 0.87*μ*; hence the necessity of a uniform temperature in the inside brass box.

### Microscopes

The microscopes have a magnifying power of very nearly 50 diameters, the objective and eyepiece each contributing equally to the power. The objectives were of the compound type, the illumination being secured by mounting small prisms in the principal focus of the lower lens of the objective. Diffused light from incandescent lamps was thrown through a screen of thin ground glass upon the prisms, from which the light was reflected vertically downward on the meter bars.

The micrometer screws were carefully studied for periodic errors about ten years ago, and a number of determinations of the screw values have been made since. They were again determined in August, 1903, and the values found were used to reduce the observations made at this period.

The values for one turn of the micrometer screws at different dates are tabulated below:

**Table:** 

Date.	Micrometer No. 6.	Micrometer No. 5.
		
	*μ*	*μ*
September–October, 1893....	75.99	74.69
July, 1894.................	.98	.67
May–September, 1896.......	.97	.76
August, 1903................	.99	.66

The foregoing values indicate a very satisfactory agreement in the screw values at widely different dates. Only one, namely, that of micrometer No. 5, made in 1896, shows an appreciable deviation from the others. The reason for this unusually large value is not known, nor is it important for the present purpose.

### Observations

An observation consisted of the following operations, which consumed about fifteen minutes:

1. Reading of thermometers in inner case.
2. Reading on No. 21.
3. Reading on No. 27.
4. Reading on No. 21.
5. Reading on No. 27.
6. Reading on No. 21.
7. Reading of thermometers in inner case.

Every time a bar was brought under the microscope the micrometers were read simultaneously four times, the observers exchanging places after the second reading to eliminate personal equation.

To insure thermal equilibrium at the beginning of the observations at least three hours was allowed to elapse between the observations.

A series consisted of eight observations, with the bars occupying as many different positions with respect to the observer and the microscopes. The positions were as follows:

**Table:** 

*First position*	*Third position*
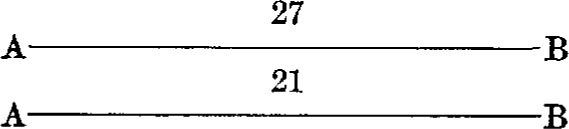	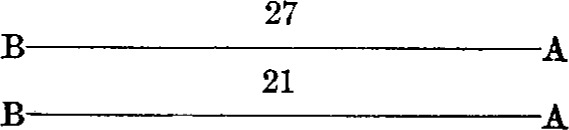
*Second position*	*Fourth position*
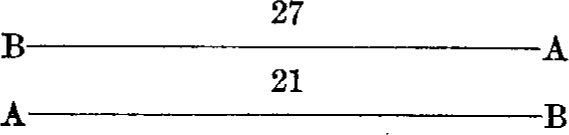	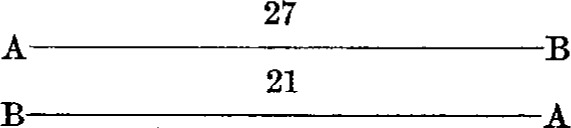

The other four positions were obtained by substituting one bar for the other in each of the above diagrams.

By this procedure each bar was in front, and hence nearer to the observer during half of the observations; also each end of both bars was brought under each microscope twice. The result of a series of observations is, therefore, independent of any possible affect of peculiar conditions.

The observations were all reduced to zero centigrade by means of the differential expansion deduced from the equations of the meter bars as furnished by the International Bureau.

In the comparison now described three series of observations were made, making a total of twenty-four observations. The results will be found in the following table, in which the first column gives the number and the second the date of the observation. The third gives the reading of the left-hand microscope on No. 21; the fourth gives the reading of the same microscope, on No. 27; the fifth gives the difference, in revolutions, of the readings of the micrometer; the sixth, the difference in microns. The seventh column gives the reading of the right-hand microscope on No. 21; the eighth, the reading of the same microscope on No. 27; the ninth, the difference in the readings of the micrometers; the tenth, the difference in microns; the eleventh, the sum of the differences of the two microscopes, in microns; the twelfth gives the mean corrected reading of the two thermometers, and the thirteenth gives the residuals for the individual observations when referred to the mean temperature of observation, namely, 23.50° C. All of the observations at the Bureau of Standards were made with the bars in air.

**Table:** First Comparisons Between Nos. 21 and 27.

Observation.	Date, 1903.	No. 6.—1 revolution=75.99*μ*.				No. 5.—1 revolution=74.66*μ*.				27–21.	Mean temperature, C.	Residuals. Obs.–cal.
		No. 21.	No. 27.	Difference in revolutions.	Difference in microns.	No. 21.	No. 27.	Difference in revolutions.	Difference in microns.			
												
1	Aug. 27	16.979	16.939	−0.040	−3.04	18.386	18.404	+0.018	+1.34	−4.38	24.127	−0.53
2	28	16.417	16.355	−0.062	−4.71	17.799	17.797	−0.002	−0.15	−4.56	24.136	−0. 71
3	28	15.872	15.865	−0.007	−0.53	17.238	17.277	+0.039	+2.91	−3.44	24.225	+0.41
4	29	15.886	15.989	+0.103	+7.83	17.256	17.412	+0.156	+11.65	−3.82	24.200	+0.03
5	29	18.094	18.176	+0.082	+6.23	19.458	19. 582	+0.124	+9.26	−3.03	24.250	+0.82
6	30	18.769	18.717	−0.052	−3.95	20.097	20.105	+0.008	+0.60	−4.55	23.701	−0.70
7	30	18.818	18.824	+0.006	+0.46	20.150	20.212	+0.062	+4.63	−4.17	23.639	−0.32
8	Sept. 1	18.789	18.732	−0.057	−4.33	19.840	19.848	+0.008	+0.60	−4.93	22.915	−1.08
9	1	18.410	18.396	−0.014	−1.06	19.706	19.740	+0.034	+2.54	−3.60	22.876	+0.25
10	2	18.315	18.256	−0.059	−4.48	19.613	19.606	−0.007	−0.52	−3.96	22.699	−0.11
11	2	18.226	18.312	+0.086	+6.53	19.516	19.658	+0.142	+10.60	−4.07	22.690	−0.22
12	3	18.124	18.079	−0.045	−3.42	19.410	19.402	−0.008	−0.60	−2.82	22.636	+1.03
13	3	19.413	19.456	+0.043	+3.27	20.705	20.798	+0.093	+ 6.94	−3.67	22.669	+0.18
14	4	19.522	19.595	+0.073	+5.55	20.849	20.968	+0.119	+8.88	−3.33	22.784	+0.52
15	5	19.756	19.880	+0.124	+9.42	21.084	21.254	+0.170	+12.69	−3.27	22.800	+0.58
16	5	19.622	19.623	+0.001	+0.01	20.960	21.010	+0.050	+3.73	−3.72	22.900	+0.13
17	6	19.854	19.750	−0.104	−7.90	20.879	20.830	+0.049	−3.66	−4.24	22.806	−0.39
18	6	19.406	19.390	−0.016	−1.22	20.428	20.456	+0.028	−2.09	−3.31	22.758	+0.54
19	7	19.504	19.376	−0.128	−9.73	20.536	20.452	−0.084	−6.27	−3.46	22.604	+0.39
20	7	19.614	19.597	−0.017	−1.29	20.629	20.658	+0.029	+2.16	−3.45	22. 510	+0.40
21	8	19.452	19.411	−0.041	−3.12	20.442	20.466	+0.024	+1.79	−4.91	22.422	−1.06
22	8	19.385	19.359	−0.026	−1.98	20.372	20.403	+0.031	+2.31	−4.29	22.380	−0.44
23	9	20.075	20.003	−0.072	−5.47	21.092	21.074	−0.018	−1.34	−4.13	22.176	−0.28
24	9	20.209	20.195	−0.014	−1.06	21.233	21.264	+0.031	+2.31	−3.37	22.174	+0.48
										−3.85	23.04	
										± 0.04	6	

Hence No. 27—No. 21=−3.85*μ* at 23.°05 C.

= −3.67*μ* at 0.°0 C.

The relation of the two bars, as determined in 1888, was

No.27−No.21=−4.00μat0.°0C.

If we assume that the length of No. 27 has remained constant, then No. 21 shows a decrease in length of 0.33*μ*. The evidence is, however, too meager to draw reliable conclusions from, and hence a discussion of this question is postponed until further observations have been made.

After the above observations had been completed, No. 27 was carefully packed in its case and transported to the International Bureau of Weights and Measures. During the transportation the bar was handled with the greatest care and received no shocks, nor was it subjected to any sudden changes of temperature. Upon the arrival of No. 27 at the International Bureau of Weights and Measures, it was immediately placed in the Brunner comparator with meter No. 26, and M. Maudet, one of the assistants at the International Bureau, was delegated to assist in the comparisons, which were begun the following day.

## The Brunner Comparator

The essential features of the Brunner comparator, which is fully described in Vol. 4, Travaux et Mémoires of the International Bureau, are two massive stone piers on which are mounted the two micrometer microscopes, and a double-walled box in which are placed the bars and which can be displaced laterally so as to bring the ends of the two standards successively under the microscopes. The trough is supported on a foundation which is only connected to the microscope piers through the earth. None of the piers are in contact with the floors of the laboratory. All the comparisons at the International Bureau were made with the bars submerged in water, which was thoroughly stirred before each observation. The temperature of the water and of the bars was determined by means of four symmetrically disposed Tonnelot thermometers, which had been used in the 1888 comparisons. These thermometers, numbered 4246, 4247, 4248, and 4249, respectively, are perhaps better known in terms of the hydrogen scale than any other mercury thermometers in existence, though in this case, as before stated, the actual temperature was of little importance.

### Micrometers

The, micrometers used are the regular micrometers belonging to the Brunner comparator, and they will be found described in the volume above referred to. Their values have been carefully determined from time to time, and the values adopted for this period by the International Bureau were used. Merely as a check a determination of the values was made by the writer after the observations had been completed, and the results obtained agreed almost perfectly with those used.

## Standards of the International Bureau

The comparison of No. 27 was made with the two standards of the International Bureau, namely, No. 26 and T_3_.

Meter No. 26, which is the principal standard, was included in the 1888 comparisons, and it was again directly compared with the international meter in 1892. T_3_, which is the secondary standard, was compared directly with the international meter in 1892, and a number of times with No. 26 between 1892 and 1894.

It might be well at this point to give the equations of all of the bars involved in the comparisons, both at the Bureau of Standards at Washington, and at the International Bureau of Weights and Measures.

They are as follows:

**Table:** 

	*μ*	*μ*	*μ*
No. 21 = 1m + 2.45 + 8.665T + 0.00100T^2^			
No. 27 = 1m − 1.55 + 8.657T + 0.00100T^2^			
= 1m − 1.55 + 8.606t + 0.00170t^2^			
No. 26 = 1m + 0.80 + 8.596t + 0.00170t^2^			
T_3_ = 1m + 1.50 + 8.583t + 0.00170t^2^			

The corrections to Nos. 21 and 27 at 0° C. are given one place further than the value furnished in their certificates, the values in the certificates having been rounded off to the nearest tenth of a micron. It was deemed advisable, in view of the small difference that might be looked for, to take the actual values found in 1888 to the nearest 0.01 of a micron. For the same reason the values of No. 26 and T_3_ were taken to the nearest 0.01 of a micron. The above values of No. 26 and T_3_ are the values at present accepted by the International Bureau as the result of the comparisons made in 1892 and 1894. In the first two equations T means temperature in degrees of the hydrogen scale. In the last three equations t means temperature in degrees of the hard glass scale of the International Bureau.^a^ In the observation made at Washington the thermometer readings were reduced to the hydrogen scale, while the observation made at the International Bureau was referred to the hard glass temperature scale. The second equation of No. 27 was therefore used in the latter observations.

## Observations

Following the method in use at the International Bureau, an observation made there consisted of five pointings on one bar and four on the other, the pointings being alternately on the two, but always beginning and ending on the same bar. A series of observations was similar to the series described in connection with the Washington observations, namely, it consisted of eight observations with the bars occupying as many different positions with respect to the observers and microscope.

The observers in these observations worked independently—that is to say, for any given position of the bars an observation was made at different times by each of the observers, the observations succeeding one another by intervals of half an hour or more. This short interval was made possible by reason of the bars being immersed in water. Two series of observations between Nos. 26 and 27 and one series between No. 27 and T_3_ were made by each observer between the 1st and the 12th of October, 1903. The results of these observations are summarized on pages 12 and 13. Below is a record of the first observation made on October 1. It is given here simply to show how the observations were made and recorded.

**Table:** Comparison of Meters Nos. 26–27.

Oct. 1, 1903. Observ: Maudet.	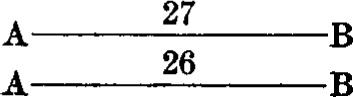

**Table:** 

**OBSERVATION.** *Thermometers.*				
4246	4247	4248	4249	Means.
16.570	16.755	16.705	16.785	16.704
.590	.760	.710	.790	.712
16.580	16.758	16.707	16.788	16.708
corr +.161	+.005	.049	−.035	.045
16.741	16.763	16.756	16.753	16.753

**Table:** 

Meter.	*Microscope.*				*Differences.*	
	Left.		Right.		Left.	Right.
27	14.773		15.067		−0.294	
26		14.825		15.088		−0.263
27	.839		.127		−0.288	
26		.772		.044		−0.272
27	.829		.123		−0.294	
26		.759		.028		−0.269
27	.839		.128		−0.289	
26		14.753		15.024		−0.271
27	14.849		15.139		−0.290	
						
	14.8258	14.7773	15.1168	15.0460	−0.2910	−0.2687
	rev.		rev.		rev.	
	+0.0485		+0.0708		−0.0223	
	+4.83*μ*		+7.10*μ*		−2.27*μ*	

**Table:** Summary of Comparison by Maudet.

First series. (27–26.)				Second series. (27–26.)				Third series. (27–T_3_.)			
Date.	Temperature.	Difference.	0-C.	Date.	Temperature.	Difference.	0-C.	Date.	Temperature.	Difference.	0-C.
											
1903.	°	*μ*	*μ*	1903.	°	*μ*	*μ*	1903.	°	*μ*	*μ*
Oct. 1	16.753	−2.27	+0.24	Oct. 5	17.001	−2.88	−0.13	Oct. 10	16.931	−3.06	+0.01
Oct. 1	.880	−2.85	−0.34	Oct. 5	.097	−2.80	−0.03	Oct. 10	.945	−3.34	−0.27
Oct. 1	.921	−2.54	−0.03	Oct. 5	.135	−3.04	−0.29	Oct. 12	.409	−2.94	+0.13
Oct. 2	.890	−2.21	+0.31	Oct. 6	.060	−2.67	+0.08	Oct. 12	.411	−3.06	+0.01
Oct. 2	17.014	−2.58	−0.07	Oct. 6	.134	−2.65	+0.10	Oct. 9	17.035	−2.77	+0.30
Oct. 2	.031	−2.43	+0.08	Oct. 6	.165	−2.56	+0.19	Oct. 10	16.924	−2.69	+0.38
Oct. 3	16.965	−2.68	−0.17	Oct. 6	.191	−2.89	−0.14	Oct. 10	.922	−3.27	−0.20
Oct. 3	17.065	−2.54	−0.03	Oct. 7	17.072	−2.51	+0.25	Oct. 10	.924	−3.42	−0.35
	16.940	−2.51	+0.64		17.107	−2.75	+0.54		16.813	−3.07	+0.83
			−0.63				−0.59				−0.82

**Table:** 

*Computation.*		
*μ*	*μ*	*μ*
(27–26) = −2.51	(27–26) = −2.75	(27–T_3_) = −3.07
26 = 1^m^+0.80	26 = 1^m^+0.80	T_3_ =1^m^+1.50
Reduction to 0° temp. = −0.17	= −0.17	= −0.39
No. 27 = 1^m^−1.88	No. 27 = 1^m^−2.12	No. 27 = 1^m^−1.96

Mean value of No. 27=1^m^−1.99*μ* at 0.°0.

**Table:** Summary of Comparison by Fischer.

First series. (27–26.)				Second series. (27–26.)				Third series. (27–T_3_.)			
Date.	Temperature.	Difference.	0-C.	Date.	Temperature.	Difference.	0-C.	Date.	Temperature.	Difference.	0-C.
											
	°	*μ*	*μ*	1903.	°	*μ*	*μ*	1903.	°	*μ*	*μ*
Oct. 1	17.091	−2.82	−0.21	Oct. 5	17.022	−2.62	+0.02	Oct. 8	17.081	−3.06	+0.08
Oct. 1	16.857	−2.51	+0.10	Oct. 5	.063	−2.78	−0.14	Oct. 8	.113	−3.30	−0.16
Oct. 1	16.937	−2.87	−0.26	Oct. 5	.162	−2.71	−0.07	Oct. 8	.143	−3.37	−0.23
Oct. 2	16.931	−2.85	−0.24	Oct. 5	.146	−2.57	+0.07	Oct. 8	.162	−2.81	+0.33
Oct. 2	16.981	−2.25	+0.36	Oct. 7	.111	−2.78	−0.14	Oct. 9	.056	−3.04	+0.10
Oct. 2	17.056	−2.31	+0.30	Oct. 7	.155	−2.39	+0.25	Oct. 9	.083	−2.94	+0.20
Oct. 3	17.007	−2.56	+0.05	Oct. 7	.185	−2.82	−0.18	Oct. 9	.119	−3.47	−0.33
Oct. 3	17.043	−2.74	−0.11	Oct. 7	17.082	−2.49	+0.05	Oct. 9	17.131	−3.11	+0.03
	16.988	−2.61	+0.81		17.116	−2.64	+0.49		17.111	−3.14	+0.74
			−0.82				−0.53				−0.72

**Table:** 

*Computation.*		
*μ*	*μ*	*μ*
(27–26) = −2.61	(27–26) = −2.64	(27–T_3_) = −3.14
26 = 1^m^+0.80	26 = 1^m^+0.80	T_3_ =1^m^+1.50
Reduction to 0° temp. = −0.17	= −0.17	= −0.39
No. 27 = 1^m^−1.98	No. 27 = 1^m^−2.01	No. 27 = 1^m^−2.03

Mean value of No. 27 = 1^m^−2.01*μ* at 0. °0.

The values deduced from the six series are remarkably concordant, the total range being only 0.24*μ*. Further, the means of the four results depending upon No. 26 agree perfectly with the two results deduced from the observation between No. 27 and T_3_. The question, therefore, of the relative weight of the values of No. 27 deduced from No. 26 and T_3_ is not important. The six results were, consequently, given equal weight. The probable error of a single observation is substantially the same for both observers; namely, 0.15*μ*. The probable error of the mean of the three series of each observer, 0.03*μ*.

For comparison the results of the two observers are repeated:

**Table:** 

*μ*
(Maudet) No. 27 = 1^m^ −1.99 at 0.°0.
(Fischer) No. 27 = 1^m^−2.01 at 0.°0.
Mean No. 27 = 1^m^−2.00 at 0. °0.

The computed probable error of the last result is ±0.02. The value originally found for No. 27 in 1888 was:

$$\text{No}.\;27=1^{m}-1.55\mu \;at\;0{}^{\circ}\;C.$$

The recent comparisons made at the International Bureau, therefore, show an apparent shortening of 0.45*μ* in the length of. No. 27 with respect to the international meter, as represented by meters No. 26 and T_3_. If the recent observations were the only evidence, there would be little doubt that a slight change had occurred in the length of No. 27, as only one observation of the 48 made gave a result as large as that previously assigned to No. 27, and moreover the probable error of the new result does not admit of a possible error greater than 0.10*μ*. On the other hand, irregular changes equally as large have been observed in other bars compared at different times at the International Bureau. The discrepancies referred to were likewise much greater than would be expected from the agreement of the observation upon which they depended.^a^

The same phenomenon has been observed in the comparison of the British imperial yard with its four copies.^b^ These bars were compared in 1855, 1876, 1882, 1892, and 1902, so that their constancy relative to one another can be studied. On account of the coarse lines and other defects in the bars from a metrological standpoint, rather large variations might be expected, but those observed are too large to be accounted for in this manner.

The question naturally arises at this point as to whether the observed differences represent real changes in the lengths of the standards, or whether they are due to certain peculiar conditions of the observers and comparing apparatus. Leaving out of consideration the yards referred to, because they belong in an entirely different class, it may be said that if the lines on the standard meters were absolutely perfect and of the same width, and if, furthermore, the surfaces on which they are ruled were uniform, and in every case perpendicular to the axes of the bars, the method of observing would eliminate any possibility for a constant error in the final result.

The lines, however, are not perfect, but the edges appear slightly ragged, under the magnifying power used, and hence the estimation of the centers of the lines is dependent to some extent upon the variable judgment of the observer. Moreover, the surfaces are not in every case exactly perpendicular to the axes of the bar, and, in consequence, the illumination of the lines is not always perpendicular to the surfaces even if the microscopes of the comparator are in perfect adjustment. Undoubtedly, part of the slight differences noted are due to the defects pointed out, but to the writer it does not appear improbable that slight temporary differences are also due to the previous history of the meters, especially to their previous thermal history.

It was the original intention of the International Committee on Weights and Measures to recompare the various national prototypes with one another and with the international meter every ten years, but this plan has not been carried out, though preparations are now being made at the International Bureau to do this work. When this has been done, it will be interesting to see whether similar changes will be observed in other prototypes, in which case it will be time to speculate as to the causes.

## Compaeison of No. 27 After Its Return to Washington

A second comparison between No. 21 and No. 27 was made in April, 1904, the result showing conclusively that the length of No. 27 had not been altered appreciably by its transportation to and from Paris. The results of these observations will be found in the following table, which is arranged in the same manner as the observations made prior to taking No. 27 to Paris. The same apparatus was used under identical conditions as in the first series of comparisons, the only difference being that the observations in this comparison were made by the writer alone.

18364–No. 1–05—2

**Table:** Second Comparison Between Nos. 21 and 27.

Observation	Date, 1904.	No. 6—1 revolution=75.93*μ*.				No. 5—1 revolution=74.8*μ*.					Mean temperature, C.	Residuals. Obs.–cal.
		No. 21.	No. 27.	Difference in revolutions.	Difference in microns.	No. 21.	No. 27.	Difference in revolutions.	Difference in microns.	27–21.		
												
1	Apr. 16	18.800	18.802	+0.002	+0.15	19.076	19.129	+0.053	+3.96	−3.81	15.619	+0.09
2	Apr. 16	18.460	18.402	−0.058	−4.41	18.712	18.707	−0.005	−0.37	−4.04	15.674	−0.14
3	Apr. 17	19.167	19.160	−0.007	−0.53	19.399	19.439	+0.040	+2.99	−3.52	15.238	+0.38
4	Apr. 17	19.263	19.218	−0.045	−3.42	19.487	19.491	+0.007	+0.52	−3.94	15.171	−0.04
5	Apr. 18	18.990	18.843	−0.147	−11.17	19.217	19.122	−0.095	−7.09	−4.08	15.146	−0.18
6	Apr. 18	18.863	18.834	−0.029	−2.20	19.092	19.114	+0.022	+1.64	−3.84	15.268	+0.06
7	Apr. 18	19.478	19.453	−0.025	−1.90	19.729	19.760	+0.031	+2.31	−4.21	15.475	−0.31
8	Apr. 18	19.531	19.467	−0.064	−4.86	19.793	19.784	−0.009	−0.67	−4.19	15.458	−0.29
9	Apr. 19	19.356	19.254	−0.102	−7.75	19.638	19.588	−0.050	−3.73	−4.02	15.432	−0.12
10	Apr. 19	19.229	19.175	−0.054	−4.10	19.497	19.495	−0.002	−0.15	−3.95	15.662	−0.05
11	Apr. 19	19.586	19.534	−0.052	−3.95	19.850	19.844	−0.006	−0.45	−3.50	15.741	+0.40
12	Apr. 19	19.698	19.688	−0.010	−0.76	19.948	19.991	+0.043	+3.21	−3.97	15.542	−0.07
13	Apr. 20	19.554	19.506	−0.048	−3.65	19.783	19.784	+0.001	+0.07	−3.72	15.462	+0.18
14	Apr. 20	19.289	19.241	−0.048	−3.65	19.488	19.491	+0.003	+0.22	−3.87	15.577	+0.03
										−3.90	15.462	
										±0.04		

No. 27–No. 21 = −3.90*μ* at 15.°462 =−3.78*μ* at 0.°0C. The result obtained prior to comparison at Paris: No. 27–No. 21=−3.67*μ* at 0.°0 C. The mean of the two results given: No. 27–No. 21=−3.72*μ* at 0.°0 C. The 1888 comparison: No. 27–No. 21=−4.00*μ* at 0.°0 C. Difference: −0.28*μ*

The two comparisons made between No. 21 and No. 27 agree so well that the conclusion is justified that No. 21 is shorter with respect to No. 27 than has been previously assumed, though the magnitude of the change is not so certain. It is nevertheless interesting to see what the change in No. 21 is on the assumption that the new value of No. 27 represents its present relation to the international meter. For this purpose the values deduced from the recent comparisons of the International Bureau and at the Bureau of Standards are summarized below:

**Table:** 

No. 21 = 27 + 3.72*μ*
No. 27 = 1^m^−2.00*μ*
hence..No. 21 = 1^m^ + l.72*μ*
Old value (1888)..No. 21 = 1^m^ + 2.45*μ*
Difference = 0.73*μ*

While the results of the new comparisons differ but slightly from the old, and are moreover not conclusive, they nevertheless introduce an uncertainty as to the lengths of Nos. 27 and 21 and make it desirable to recompare No. 27 directly with the international and other national prototypes. Until this has been done by the International Bureau the old value of No. 27 will be used.

It is probable that part of the differences observed are due to slight errors in the coefficients of expansion of No. 27 and No. 21, and in order to test this, and also to fix definitely for this period the relative values of these bars, further comparisons will be undertaken at once.

In conclusion, the writer wishes to express his obligations to Dr. Benoît, director, and Dr. Guillaume, assistant director, respectively, of the International Bureau of Weights and Measures, for placing at his disposal all the required apparatus of the Bureau and for many valuable suggestions; to M. Maudet, who shared equally in the work at the International Bureau, and who made most of the reductions; and also to Mr. L. G. Hoxton, of the Bureau of Standards, who assisted in the first series of observations made at Washington prior to the comparison at Paris.

## Figures and Tables

**Fig. 1:**
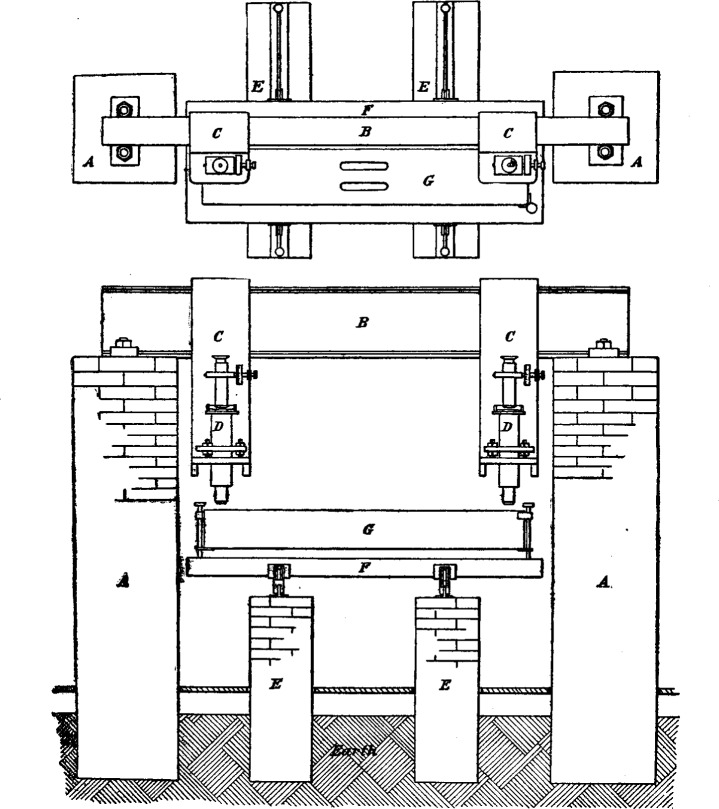
Arrangement of the comparator.

## Footnotes

a For a full description of the prototype meter, its transportation and acceptance, see Appendix 18, U. S. Coast and Geodetic Survey Report for 1890.

a Nouvelles déterminations des Metres Étalons: Travaux et Mémoires, Vol. 11, p. 6.

a Nouvelles déterminations des Metres Étalons: Travaux et Mémoires, Vol. 11, p. 20.

b An Account of the Comparison of Four Parliamentary Copies of the Imperial Standards, 1902.

